# Overexpression of OsHMGB707, a High Mobility Group Protein, Enhances Rice Drought Tolerance by Promoting Stress-Related Gene Expression

**DOI:** 10.3389/fpls.2021.711271

**Published:** 2021-08-05

**Authors:** Kai Xu, Shoujun Chen, Tianfei Li, Shunwu Yu, Hui Zhao, Hongyan Liu, Lijun Luo

**Affiliations:** ^1^Shanghai Agrobiological Gene Center, Shanghai, China; ^2^College of Plant Science and Technology, Huazhong Agricultural University, Wuhan, China

**Keywords:** gene expression regulation, high mobility group protein, rice, OsHMGB707, drought tolerance

## Abstract

Drought stress adversely affects crop growth and productivity worldwide. In response, plants have evolved several strategies in which numerous genes are induced to counter stress. High mobility group (HMG) proteins are the second most abundant family of chromosomal proteins. They play a crucial role in gene transcriptional regulation by modulating the chromatin/DNA structure. In this study, we isolated a novel HMG gene, *OsHMGB707*, one of the candidate genes localized in the quantitative trait loci (QTL) interval of rice drought tolerance, and examined its function on rice stress tolerance. The expression of *OsHMGB707* was up-regulated by dehydration and high salt treatment. Its overexpression significantly enhanced drought tolerance in transgenic rice plants, whereas its knockdown through RNA interference (RNAi) did not affect the drought tolerance of the transgenic rice plants. Notably, OsHMGB707-GFP is localized in the cell nucleus, and OsHMGB707 is protein-bound to the synthetic four-way junction DNA. Several genes were up-regulated in OsHMGB707-overexpression (OE) rice lines compared to the wild-type rice varieties. Some of the genes encode stress-related proteins (e.g., DREB transcription factors, heat shock protein 20, and heat shock protein DnaJ). In summary, *OsHMGB707* encodes a stress-responsive high mobility group protein and regulates rice drought tolerance by promoting the expression of stress-related genes.

## Introduction

Drought and water defects are key abiotic stresses that adversely affect agricultural production (Torres and Henry, 2007). Rice is one of the most important crops globally in terms of production and consumption, alongside wheat and maize. However, rice production requires more water input than most major crops like wheat and maize (Luo, 2010). Elucidating the molecular mechanism underlying the drought tolerance in rice and deploying water-saving and drought-resistant rice varieties are effective methods for reducing crop production loss brought by frequent water shortages and drought stress.

To enhance efficient water absorption and withstand drought stress, plants have adopted various strategies like osmotic adjustment, accumulating antioxidants, inducing stomatal closure (to reduce water loss), and developing a deep root system (Dorothea and Ramanjulu, 2005; Muhammad et al., 2009). Plants sense and respond to drought stress rapidly by transducing signals to cells, which activate the expression of several genes to resist stress (Hirayama and Shinozaki, 2010). Transcription control is one of the most important steps during plant drought stress response.

Chromatin/DNA structure rearrangement and transcription initiation are vital steps of gene expression regulation (Agresti and Bianchi, 2003). High mobility group proteins (HMG) play a key role in regulating these steps. They are abundant proteins associated with chromatin (Grosschedl et al., 1994). Studies have shown that mammalian HMG proteins play an essential role in regulating gene transcription through bending, modifying, or changing the chromatin DNA structure. This benefits numerous protein components, which assemble the protein complex for transcription (Pil et al., 1993; Grosschedl et al., 1994; Lehming et al., 1994). In eukaryotes, there are three main HMG protein subfamilies, including HMGA, HMGB, and HMGN (Bianchi and Agresti, 2005). HMGB proteins were the first to be studied. They are highly mobile and abundant in the cell nucleus. Recent studies demonstrate that HMGB1 associates with various molecules, including DNA, RNA, proteins, and lipopolysaccharides, to mediate various processes in DNA metabolism and innate immunity (Mandke and Vasquez, 2019). Additionally, HMGB1 potentially contributes to all the stages of tumorigenesis (Rapoport et al., 2020).

In higher plants, several HMG genes have been isolated (mainly from Arabidopsis and maize), and their protein functions were identified (Grasser et al., 2007). Studies have shown that HMG proteins play crucial roles in plant development and abiotic stress responses (Pedersen and Grasser, 2010). For example, the overexpression of the HMGB gene *ZmHMGB1* in maize inhibited root growth (Lichota et al., 2004). AtHMGB15 promoted pollen tube development in Arabidopsis (Xia et al., 2014) and regulated the expression of stress response genes under cold stress (Mallik et al., 2020). HMGB3 also participates in the activation of plant innate immunity (Choi et al., 2016). Besides, other HMGB genes (i.e., *HMGB1*, *HMGB2*, and *HMGB5*) play distinctive roles in cellular salt or dehydration tolerance (Kwak et al., 2007; Lildballe et al., 2008). In rice, there are 11 HMG genes, including six HMGB genes. Among them, only two genes (*HMGB1* and *HMGB2*) have been cloned and characterized (Grasser et al., 2007). However, the function of rice HMGB genes in stress tolerance remains unclear.

Many drought-tolerant quantitative trait loci (QTLs) have been mapped (Zeng et al., 2006), but only a few candidate genes associated with drought QTLs have been characterized. In our lab, rice drought tolerance QTLs intervals were mapped, and through bioinformatics analysis, some candidate genes were discovered (Zou et al., 2005; Zeng et al., 2007). In this study, we isolated a novel HMGB gene (*OsHMGB707*) localized in the rice drought QTL interval region and investigated its function in drought response. Its overexpression promoted the expression of numerous stress-related genes like *OsDREB1G* and significantly improved drought tolerance in rice. To the best of our knowledge, this is the first study to explore the role of rice HMGB protein in drought tolerance. This study provides novel insights into the molecular mechanism of drought stress responses in rice.

## Materials and Methods

### Gene Isolation

In our previous study, bioinformatics and expression pattern analysis identified several candidate genes in the QTL interval of rice drought tolerance of chromosome 4 (Zou et al., 2005; Zeng et al., 2007). One of these candidate genes, *OsHMGB707* was amplified from the cDNA of the IRAT109 rice cultivar, and its PCR product cloned into the pMD18T vector and then sequenced. The resulting DNA sequence was translated into the amino acids sequence, and a multiple sequence alignment of HMG proteins from Arabidopsis and rice was performed using Clustal X. The phylogenetic tree was constructed using the neighbor method (MEGA5.0 software) (Tamura et al., 2011).

### Gene Expression Pattern Analysis

To analyze the expression pattern of *OsHMGB707*, seedlings of the rice cultivar Nipponbare (*Oryza sativa L.* ssp *japonica*) at the four-leaf stage were subjected to various treatments, including dehydration (water withholding), salt (150 mM NaCl), oxidative stress (1% H_2_O_2_), cold (4°C), and heat (42°C). Also, plant hormones like 0.1 mM abscisic acid (ABA), and jasmonic acid (JA) were separately sprayed on the seedlings. The roots were also submerged into the solution and then sampled at designated times.

Total RNA was extracted using TRNzol reagent (TIANGEN, DP424, China), and cDNA synthesized using PrimerScript reverse transcriptase (TaKaRa, RR036A, Japan). Quantitative PCR (qPCR) was performed in a 96-well plate with a Bio-Rad CFX96 Real-Time PCR Detection System (Bio-Rad, United States) using the SYBR premix Ex Taq (TaKaRa, RR820A, Japan) according to the manufacturer’s instructions. The reaction conditions were as follows: 95°C for 60 s, followed by 40 cycles at 94°C for 15 s and 62°C for 60 s. The rice actin gene *OsACT2* (*Os11g0163100*) was used as the reference gene to normalize the target gene expression, calculated using the relative quantification method (2^–ΔΔ*CT*^).

### Vector Construction, Rice Transformation, and Molecular Characterization of Transgenic Rice Plants

Full-length cDNA of *OsHMGB707* was digested with *Xba*I and *Bst*EII enzymes and then ligated into the plant expression vector pCAMBIA1323 digested using the same enzymes. Notably, *OsHMGB707* was driven by the cauliflower mosaic virus (CaMV) 35S promoter. For the RNAi vector construction, two copies of the 300 bp segment of *OsHMGB707* were inserted into the pTCK303 vector. Both the above recombinant constructs were introduced into the japonica rice Zhonghua11 (ZH11) via *Agrobacterium*-mediated transformation as described previously (Lin and Zhang, 2005). The transformed rice plants were selected on the Murashige and Skoog (MS) medium containing 50 mg/L hygromycin (Murashige and Skoog, 1962).

Subsequently, the transgenic rice plants were characterized using PCR to confirm whether *OsHMGB707* was successfully integrated into their genome. To evaluate the *OsHMGB707* expression in *OsHMGB707*-overexpressing rice plants and RNAi plants, Real Time-qPCR was performed, and its expression in transgenic rice lines calculated as described in section “Gene expression pattern analysis.”

### Stress Treatment and Physiological Index Determination

The seeds of T3 positive overexpression (OE) and RNAi lines were germinated on MS medium supplemented with 50 mg/L hygromycin for different stress treatments. Similarly, the wild-type (WT) seeds were also cultured in the MS medium without hygromycin. The most evenly germinating seeds were sown in a 96-well plate from which the bottom had been removed for osmotic stress treatment. The seedlings were grown in liquid culture solution in a growth chamber with a 16 h light (28°C)/8 h dark (24°C) photoperiod/temperature rotation. Consequently, 21 days old seedlings were transferred into liquid culture solution supplemented with 18 or 20% (m/V) polyethyleneglycol (PEG) 6000 to prompt osmotic stress for 2–3 days. After the WT plants were wilted, they were transferred into normal culture solution for 14 days, and the number of surviving plants was recorded to calculate the survival rate.

Drought tolerance testing was performed for 30 days in a greenhouse. The experiment was initiated at the panicle development stage after the water supply was stopped. After severe wilting of WT plants, all the plants were re-watered until harvest, and their agronomic traits and yield traits were measured.

Subsequently, the leaves from the plants treated with PEG were sampled for physiological analysis. The total malondialdehyde (MDA) and soluble sugar contents were measured using a commercial kit from the Nanjing Jiancheng Bioengineering Institute (Jiancheng, A003-3-1 and A145-1-1, China). For water loss rate measurements, leaves were detached from rice plants at the vegetable stage and weighed every hour.

### Sub-Cellular Localization

To examine the subcellular localization of the OsHMGB707 protein, the full-length of *OsHMGB707* was cloned into the plant expression vector pCAMBIA1300EGFP after being digested with *Xba*I and *Bam*HI, fusing OsHMGB707 to GFP. The GFP fusion vector was transformed into *A. tumefaciens* strain EHA105. The transformed *Agrobacterium* was infiltrated into the leaves of *Nicotiana benthamiana* plants as previously described (Liu et al., 2010). These agroinfiltrated plants were left to grow for 48 h. Propidium iodide (PI) was infiltrated into these leaves 3 h before observation, and the GFP and PI fluorescence was examined under a laser confocal microscope (Olympus, FV3000, Japan).

### Protein Expression and Purification

For OsHMGB707 protein expression, *OsHMGB707* was cloned into the GST expression vector pGEX-6P-1, and the GST fusion expression constructs were introduced into *E. coli BL21*. The expression of the fusion protein was induced by adding 1 mM IPTG and incubated at 18°C overnight. The solubility of the expressed protein was checked using sodium dodecyl sulfate-polyacrylamide gel electrophoresis (SDS-PAGE). We induced GST-OsHMGB707 in 100 mL LB medium to purify the fusion protein, and then bacteria were broken up using a supersonic broker (Scientz, JY92-2D, China). Lastly, the supernatant was added into Profinity GST chromatograph column (Bio-Rad, 7324624, United States) in the Profinia protein purification system (Bio-Rad, United States), and the purified protein was checked through SDS-PAGE.

### Electrophoretic Mobility Shift Assays (EMSA)

The four-way junction DNA was formed through annealing the four poly nucleic acids and then purified using PAGE as described before (Wu et al., 2003). The promoter segments (∼200 bp) were amplified using biotin-labeled primers. To analyze the OsHMGB707 DNA binding ability, EMSA assay (Hellman and Fried, 2007) was performed using a LightShift Chemiluminescent EMSA kit (Thermo Fisher Scientific, 20148, United States) following the manufacturer’s instructions. The OsHMGB707 protein and biotin-labeled DNA were added to the reaction buffer at room temperature then incubated for 20 min to assess the DNA binding reaction. After this reaction, all samples were loaded into the PAGE gel system and transferred to a Hybond-N^+^ nylon membrane (Amersham, United States). The membrane was then washed and the biotin-labeled DNA was detected by Chemiluminescence. The membrane was exposed to an X-ray film or a ChemiDoc XRS + imaging system (Bio-Rad, United States).

### Micro-Array Analysis

Twenty-one day-old seedlings of WT, *OsHMGB707*-OE, and *OsHMGB707*-RNAi rice plants were harvested for subsequent microarray analysis. Total RNA was extracted from three biological replicates (20 seedlings per replicate) using TRizol reagent. The experimental procedure followed the standard protocol of the Affymetrix GeneChip service (Gene biotech). The differentially expressed genes (DEGs) between the transgenic rice plants and WT plants samples were identified. The fold change was greater than 2 (up-regulated) or less than 0.5 (down-regulated) using the analysis software. The expression of selected DEGs was confirmed using quantitative PCR. DEGs in OsHMGB707 transgenic plants whose expression was affected by drought stress were selected based on the gene expression profile of WT plants under drought stress detected previously (The data is available in NCBI Gene Expression Omnibus repository under accession number GSE64576). A part of genes whose expression changes are opposite in RNAi plants compared with OE plants and under drought stress were chosen and the heatmap of the selected DEGs was produced using Clustvis 2.0^1^ (Metsalu and Vilo, 2015).

### Biochemical Assays in Yeast

For *trans*-activation activity assay in yeast, the CDS of OsHMGB707 was cloned into the pGBKT7 vector and were introduced into yeast strain Y2H Gold according to the manufacturer’s instruction (Clontech, 630489, United States). The *trans*-activation activity was tested through spotting the yeast on SD/Trp- His- plates containing 30 mM 3-AT and incubating at 30°C for 2 days.

For yeast one-hybrid assays, we used the Matchmaker one-hybrid system following the manual instruction (Clontech, PT1031-1, United States). We isolated four target gene promoter segments (∼200 bp) of OsHMGB707, cloned them into the yeast vector pHIS2.1 to be the reporter constructs. The CDS of OsHMGB707 was fused to the GAL4 activation domain in pGADT7-Rec2 and transformed them into the yeast Y187 strain with reporter constructs. Finally, the binding activities were tested through spotting the yeast on the SD/Leu-Trp- His- plates containing 30 mM 3-AT and incubating at 30°C for 2 days.

## Results

### Isolation and Sequence Analysis of *OsHMGB707*

**FIGURE 1:**
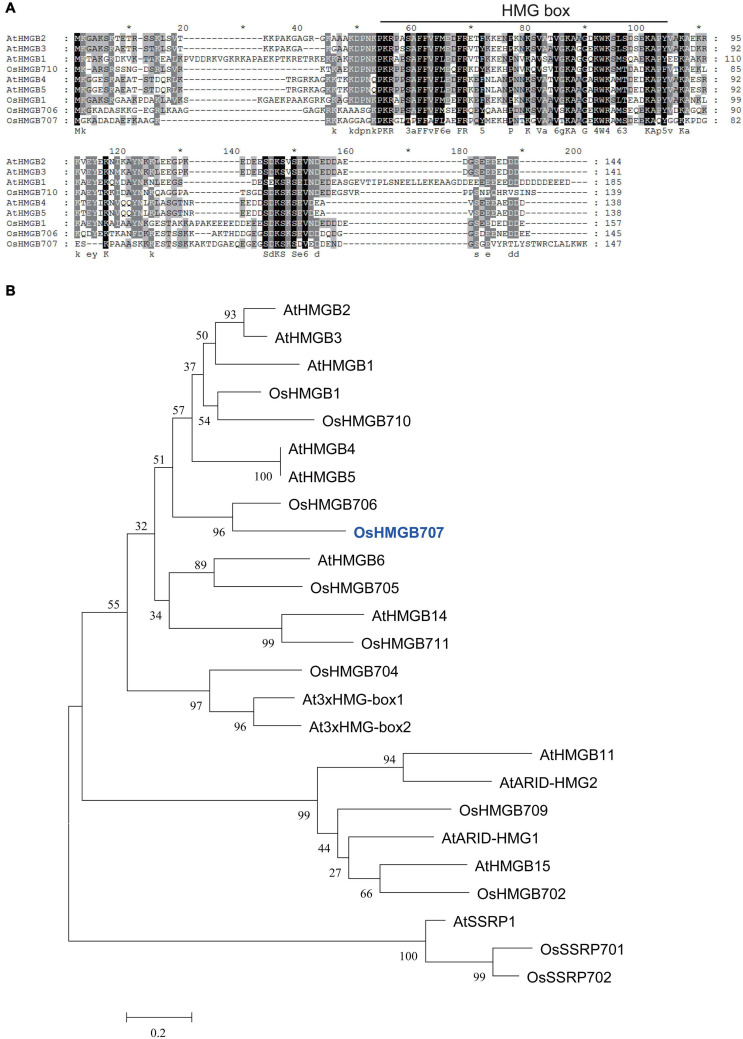
Sequence alignment and homological analysis of OsHMGB707 and other HMG proteins from Arabidopsis and rice. **(A)** Protein sequence alignment of several Arabidopsis and rice HMGB subfamily proteins. The HMG domain is indicated by lines. **(B)** Phylogenetic tree of Arabidopsis and rice HMG proteins. The tree was constructed using MEGA5.0.

In our previous study, one QTL interval in chromosome 4 that harbors a cluster conferring drought tolerance and yield traits was mapped and selected for further evaluation in the present study. Among the genes located in this QTL interval include several drought-induced genes, which were selected as candidate genes. One of these candidate genes, *OsHMGB707*, encodes an HMG protein. Its expression in parent IRAT109 is higher than Zhenshan97B (Supplementary Figure 1). This gene was cloned from the upland rice IRAT109. Of note, the coding sequence of OsHMGB707 in IRAT109 is similar to that of Nipponbare rice. The amino acid alignment showed that OsHMGB707 has high homology to AtHMGB1-5. Similar to the Arabidopsis HMGB proteins, OsHMGB707 has one conserved HMG domain (Figure 1A) and a variable C-terminal. Phylogenetic analysis showed that OsHMGB707 belongs to the HMGB subfamily, near AtHMGB1-5 (Figure 1B).

### Expression Pattern of *OsHMGB707*

**FIGURE 2:**
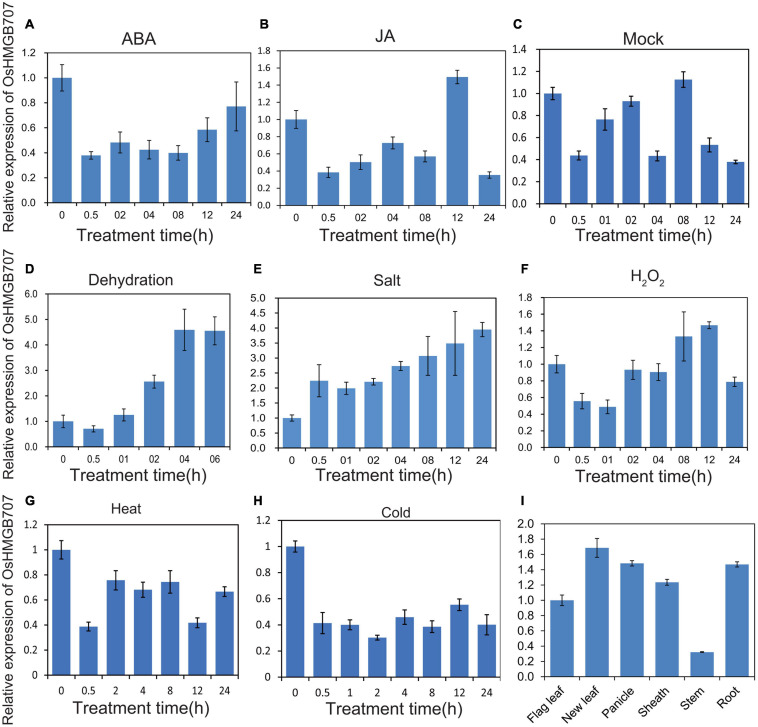
Expression pattern of *OsHMGB707*. **(A–C)** Relative expression of *OsHMGB707* under different hormone treatments (ABA, JA, Mock). **(D–H)** Relative expression of *OsHMGB707* under different stress treatments (Dehydration, high salt, H_2_O_2_, heat, and cold). Rice seedlings at the four-leaf stage were treated with 0.1 mM ABA, 0.1 mM JA, 0.1 mM GA, dehydration, salt (150 mM NaCl), oxidative stress (1% H_2_O_2_), cold (4°C), and heat (42°C). **(I)** Relative expression of *OsHMGB707* in rice tissues. The expression levels of *OsHMGB707* were analyzed using qPCR. Error bars indicate the standard error (SE) of three biological replicates.

Real-time qPCR was performed to determine the expression pattern of *OsHMGB707* under stresses and phytohormone treatments. Dehydration and salt stress treatments significantly upregulated the expression of *OsHMGB707* (Figure 2). In contrast, cold stress downregulated *OsHMGB707* expression. The expression of *OsHMGB707* reduced under ABA and JA treatments. These results suggest that *OsHMGB707* may participate in abiotic stress responses. The results of *OsHMGB707* expression in various rice tissues show that it is expressed in leaves, shoots, panicles, roots, and other tissues (Figure 2), indicating that *OsHMGB707* expression is not tissue-specific.

### Overexpression of *OsHMGB707* Enhances Osmotic Tolerance in Transgenic Rice Plants

For overexpression, *OsHMGB707* was driven by the CaMV35S promoter (Supplementary Figure 2A). Real-time qPCR analysis showed that several transgenic lines displayed higher expression of *OsHMGB707* than the WT plants (Supplementary Figure 2B).

**FIGURE 3:**
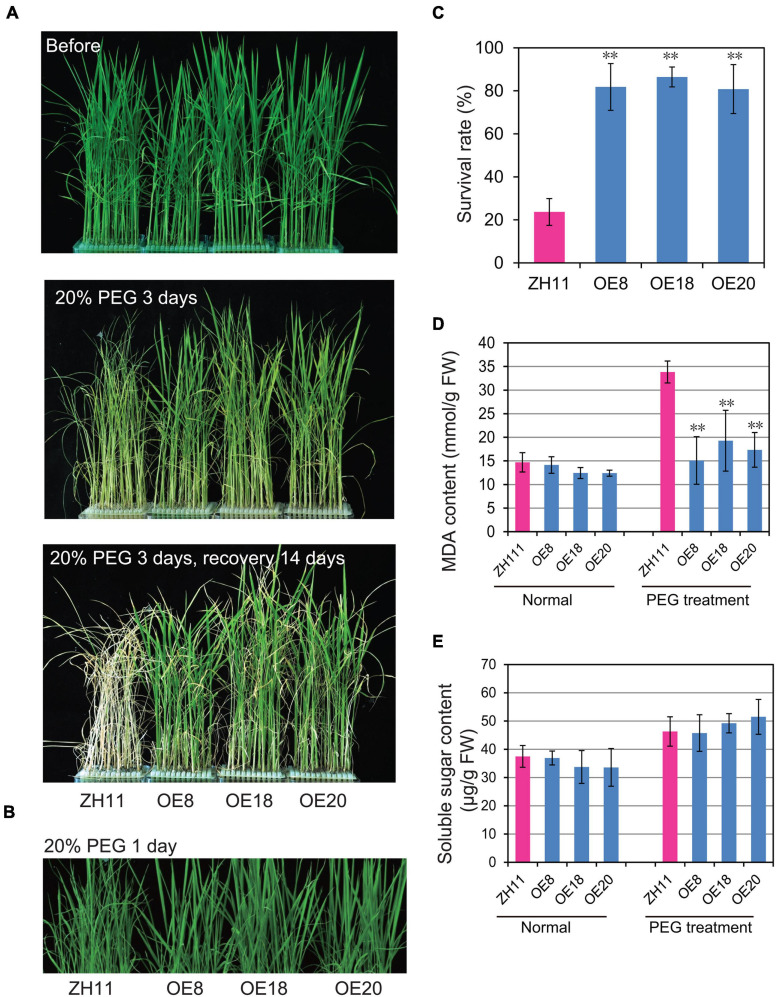
Overexpression of *OsHMGB707* improves the tolerance of transgenic rice plants to osmotic stress. **(A)** Osmotic stress treatments of *OsHMGB707*-overexpression (OE) lines and WT rice plants. Plants were treated with 20% (m/V) PEG6000 for 3 days and recovered for 14 days. **(B)** *OsHMGB707* OE plants displayed less leaf rolling when treated with 20% (m/V) PEG6000 for 1 days. **(C)** Survival rates of transgenic and WT rice plants after osmotic stress. All data were collected in three biological replicates (*n* = 48 each). Malondialdehyde (MDA) content **(D)** and soluble sugar content **(E)** *of OsHMGB707* OE lines and WT rice plants. All data were collected with three biological replicates (*n* = 10 each). Error bars indicate the standard error (SE) of three biological replicates. **p < 0.01, Student’s *t*-test.

To investigate whether *OsHMGB707* participates in rice drought tolerance, the *OsHMGB707* OE lines were treated with PEG to simulate osmotic stress. Before treatment, there was no obvious phenotypic difference between the transgenic seedlings and the WT (Figure 3A). However, the leaves of WT plants wilted rapidly 1 day post-treatment with 20%PEG (Figure 3B) and rolled adversely 2 days post-treatment with 20% PEG. After recovery treatment for 14 days, most WT plants did not survive. In contrast, *OsHMGB707* overexpression lines wilted slightly under PEG treatment and soon recovered after they were transferred to normal culture solution (Figure 3A). The survival rates of *OsHMGB707* OE rice plants (80–86%) were significantly higher than WT plants (23%) (Figure 3C).

Several drought-related physiological traits of the transgenic plants were examined under drought stress. Although the soluble sugar contents of the transgenic rice leaves were similar to the WT rice (Figure 3E), the MDA contents of transgenic lines were significantly lower than WT plants under PEG-simulate osmotic stress (Figure 3D).

### Silencing of *OsHMGB707* Does Not Significantly Affect Osmotic Stress Tolerance of Transgenic Rice Plants

Herein, we generated *OsHMGB707* RNAi lines to further assess the drought tolerance function of *OsHMGB707*. One segment of *OsHMGB707* was cloned into vector pTCK303 under the control of the ubiquitin promoter (Supplementary Figure 3A). Real-time qPCR analysis showed that *OsHMGB707* expression in several RNAi transgenic rice plants (e.g., RNAi-11and RNAi-17) was significantly lower than the WT plants (Supplementary Figure 3B).

**FIGURE 4:**
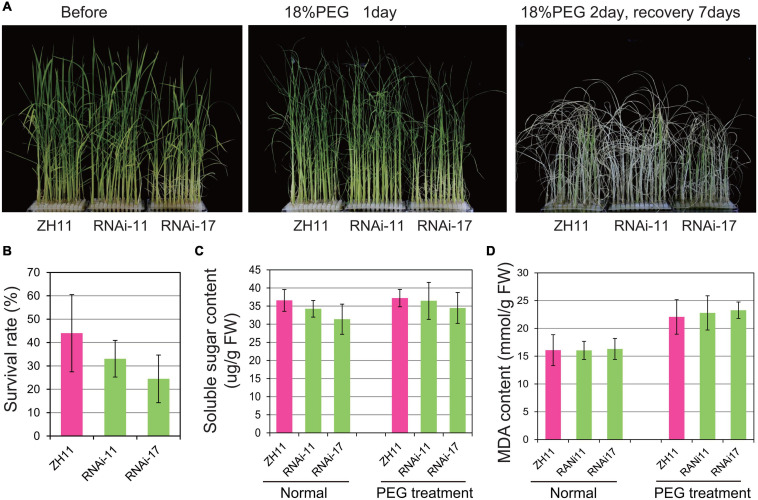
Knockdown expression of *OsHMGB707* does not significantly affect the osmotic stress tolerance of transgenic rice plants. **(A)** Osmotic stress treatments of *OsHMGB707* knockdown lines and WT plants. Plants were treated with 18% (m/V) PEG6000 for 2 days and recovered for 7 days. **(B)** The survival rates of transgenic lines and WT plants after osmotic stress. All data were collected in three biological replicates (*n* = 48 each). **(C)** Soluble sugar content and **(D)** Malondialdehyde (MDA) content of *OsHMGB707*-RNAi lines and WT rice plants. All data were collected in three biological replicates (*n* = 10 each). Error bars indicate the standard error (SE) of three biological replicates.

The *OsHMGB707* RNAi plants were further treated with PEG-simulated osmotic stress. There was no obvious phenotypic difference between the RNAi and WT rice plants before treatment. Indeed, the leaves of RNAi rice plants rolled in a pattern similar to the WT plants under PEG-simulated osmotic stress treatment for 1 day (Figure 4A). Besides, there was no significant difference between the survival rates of *OsHMGB707* RNAi plants and the WT after stress treatment (Figure 4B). Consistently, there was no significant difference in the MDA and soluble sugar contents between the *OsHMGB707* RNAi and WT plants under stress treatment (Figures 4C,D). These results indicate *OsHMGB707* knockdown does not significantly affect the osmotic tolerance of transgenic rice plants.

### Drought Tolerance of *OsHMGB707* Transgenic Rice Plants Under Field Conditions

**FIGURE 5:**
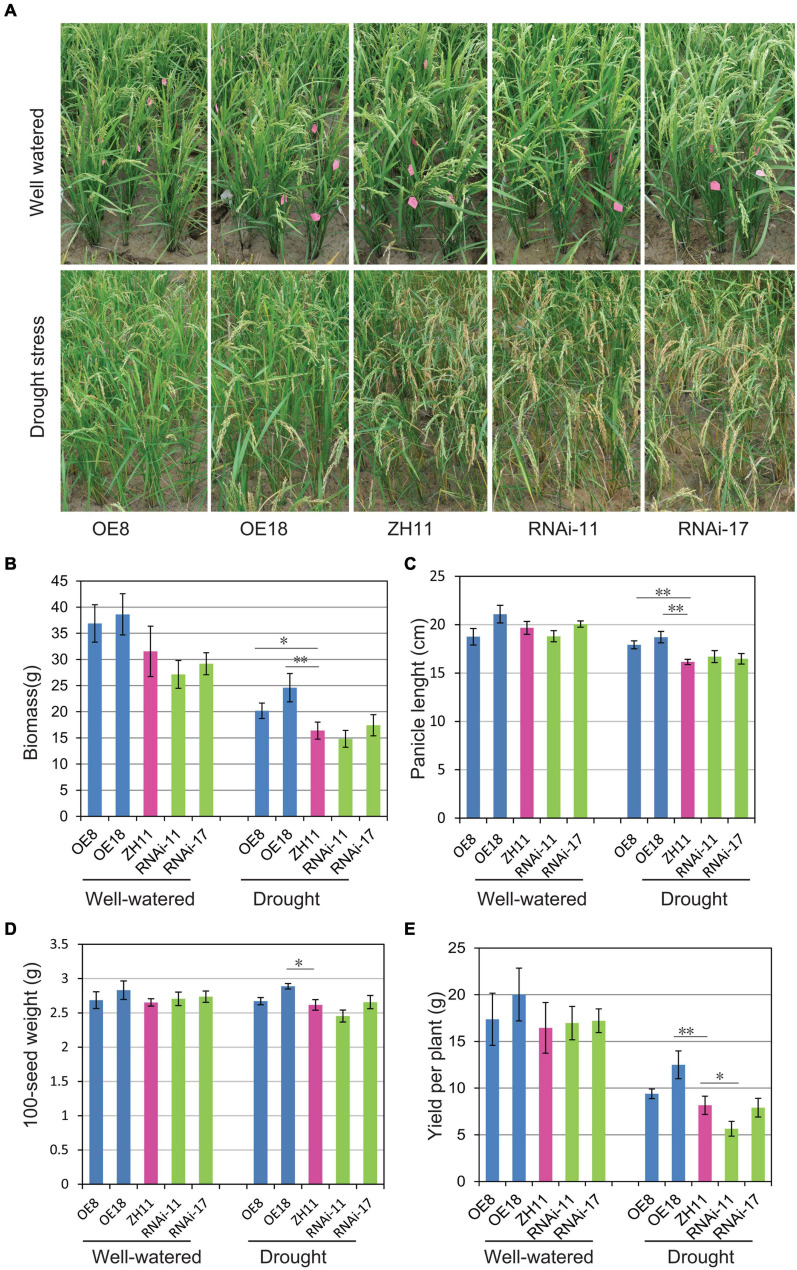
Drought tolerance assessment of *OsHMGB707* transgenic lines and WT rice plants under field conditions. **(A)** The performance of *OsHMGB707*-overexpression (OE) lines (OE8 and OE18), *OsHMGB707*-RNAi lines (RNAi-11 and RNAi-17), and WT rice plants (ZH11) before and after drought stress in the field. The rice plants were cultivated in the greenhouse, and irrigation was stopped before the heading stage. The water was re-supplied after 30-day drought treatment. **(B)** Biomass, **(C)** panicle length, **(D)** 100-seed weight, **(E)** yield per plant in transgenic lines and WT rice plants. Data represents means ± standard error (SE) (*n* = 8–10), *p < 0.05, **p < 0.01, Student’s *t*-test.

To further investigate the drought tolerance of transgenic rice plants under field conditions, the *OsHMGB707* OE, RNAi, and WT rice plants were planted in the field and subjected to drought treatment at the young panicle differentiation stage. Under drought treatment, the *OsHMGB707* OE rice plants exhibited excellent growth with fewer leaves rolled than in WT and RNAi lines. Meanwhile, the leaves of RNAi lines rolled like those of the WT (Figure 5A). Under normal conditions, there was no significant difference in the biomass and yield between the *OsHMGB707* OE lines and the WT plants. However, under drought stress, the biomass, panicle length, 100-seed weight, and yield per plant were significantly higher in *OsHMGB707* OE rice plants (line OE8 or OE18) than in WT plants. There was no significant difference in the agronomic traits between *OsHMGB707* RNAi and WT rice plants, except that the yield per plant of RNAi-11 lines was lower than WT plants (Figures 5B–E). The field test was repeated and OE plants under drought stress exhibited similar higher biomass and yield compared to WT plants (Supplementary Figure 4). These results further demonstrate that *OsHMGB707* facilitates rice drought tolerance and can therefore be exploited in breeding drought-resistant rice varieties.

This study explored several drought tolerance-related traits to further understand the mechanism for the enhanced drought tolerance in *OsHMGB707* OE rice plants. The WT tended to show higher water loss rate of the detached leaves compared to the *OsHMGB707* OE lines, but the difference was not statistically significant (Supplementary Figure 5A). The root lengths of *OsHMGB707* OE transgenic rice plants and WT plants were similar under normal and drought stress conditions (Supplementary Figure 5B).

### Sub-Cellular Localization, *Trans*-Activation, and DNA Binding Ability of OsHMGB707

**FIGURE 6:**
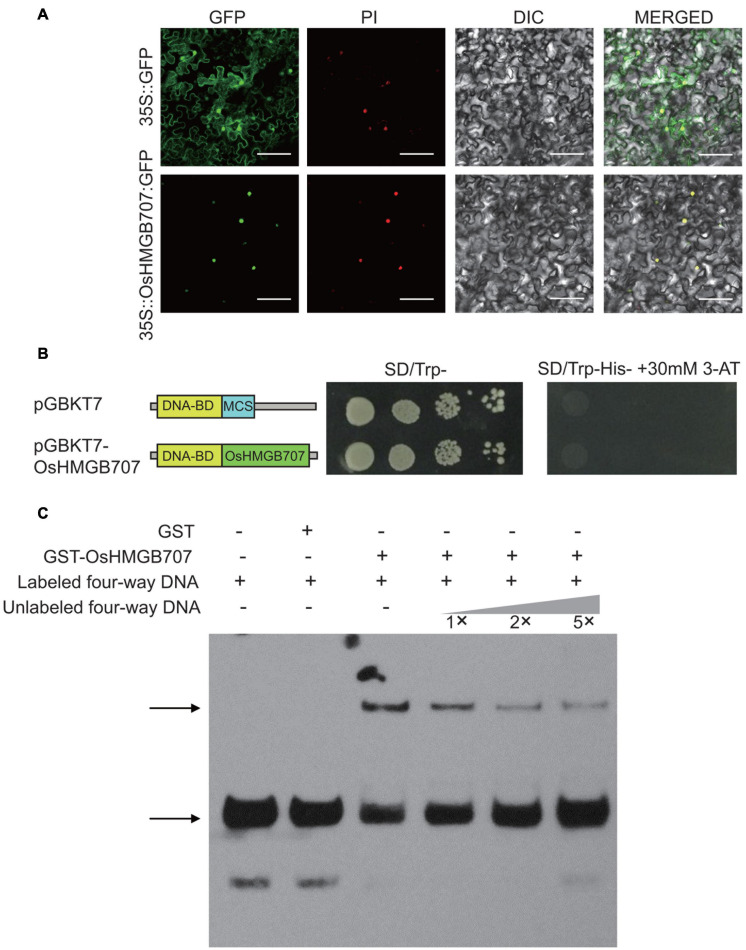
Sub-cellular localization, *trans*-activation, and DNA binding test of OsHMGB707. **(A)** OsHMGB707-GFP fusion proteins were expressed in tobacco leaf epidermal cells, and GFP fluorescence was detected using a confocal microscope. PI, Propidium Iodide; DIC, Differential Interference Contrast; MERGE, overlay of GFP, PI, and DIC images. Scale bar, 50 μm; **(B)** GAL4 BD-OsHMGB707 protein was expressed in Y2H Gold yeast cells, and the transcription activity was detected by growing on SD medium. **(C)** OsHMGB707 protein can bind to the synthetic four-way junction DNA *in vitro*, as indicated by EMSA assay. The GST-OsHMGB707 protein and biotin-labeled synthetic four-way junction DNA were reacted *in vitro* and detected through the biotin reaction. The upper arrow shows the protein-DNA binding complex, while the lower arrow indicates the free four-way junction DNA.

To investigate the subcellular localization of OsHMGB707, an OsHMGB707-GFP fusion protein was constructed and introduced to tobacco leaves. The fluorescence signals of OsHMGB707-GFP were detected in the cell nucleus of tobacco epidermal cells, merged with PI, which is a kind of nuclear stain (Figure 6A). Generally, HMG proteins have no classic transcription factor for trans-activation activities. This study also investigated the *trans*-activation activity of OsHMGB707 in yeast to verify the similar attribute of OsHMGB707. The results showed that OsHMGB707 is a cell nuclear protein with no trans-activation activity (Figure 6B).

High mobility group proteins are commonly associated with chromatins/DNA and mainly function to bind various DNA structures, including four-way junction DNA, and to aid the assembly of the transcription protein complex. To determine whether OsHMGB707 can bind DNA, the GST-OsHMGB707 was expressed in *E. coli* and purified (Supplementary Figure 6). The four-way junction DNA was produced via annealing and recycling. The EMSA assay showed that OsHMGB707 binds to the synthetic four-way junction DNA and competes with the unlabeled DNA (Figure 6C).

### Gene Expression Profiles of *OsHMGB707* OE and RNAi Transgenic Rice Plants

Microarray analysis was conducted to determine the effect of OsHMGB707 on the gene expression profiles of transgenic rice plants. A total of 107 genes were up-regulated while 88 genes were down-regulated in *OsHMGB707* OE plants, compared with the WT plants (Supplementary Table 1). Meanwhile, 39 genes were down-regulated, and 110 genes were up-regulated in RNAi plants, relative to the WT plants (Supplementary Table 2). Only one overlapped gene, *OsDREB1G*, was found between the up-regulated genes in *OsHMGB707* OE plants and down-regulated genes in RNAi plants, suggesting that *OsDREB1G* might be the target gene of OsHMGB707.

**FIGURE 7:**
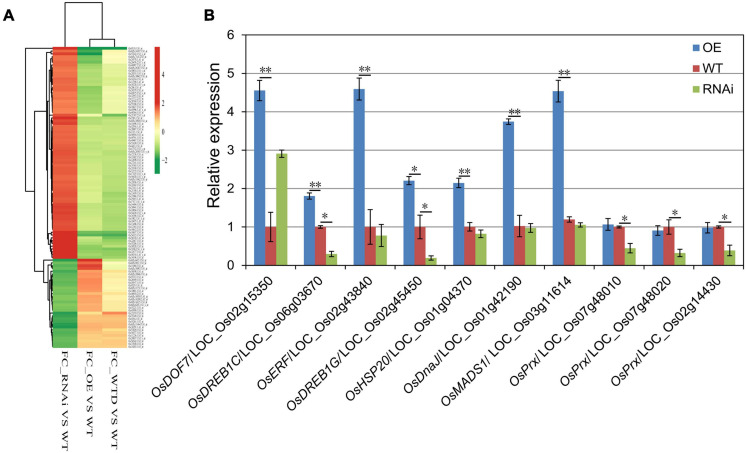
The gene expression profiles of *OsHMGB707* transgenic rice plants. **(A)** Cluster analysis of selected DEGs between transgenic lines and WT rice plants. FC, fold change; WTD, WT plants under drought stress; WT, WT plants under normal conditions. **(B)** qPCR analysis to validate the expression of some DEGs. Error bars indicate the standard error (SE) of three biological replicates, *p < 0.05, **p < 0.01, Student’s *t*-test.

Some of the up-regulated DEGs in OE plants were also drought-induced, compared with the micro-array data of zhonghua11 under drought treatment (Figure 7A). Besides, some of these up-regulated genes encode stress-related transcription factors and heat shock proteins. For instance, *LOC _Os02g45450 (OsDREB1G)* and *LOC_Os06g03670* (*OsDREB1C*) encode dehydration-responsive element-binding protein (DREB), *LOC_Os02g43840* encodes an ethylene-responsive element-binding protein (ERF), *LOC_Os01g42190* encodes the heat shock protein DnaJ, and *LOC_Os01g04370* encodes the heat shock protein 20. Several down-regulated genes in *OsHMGB707*-RNAi plants encode peroxidases (Prx) (e.g., *LOC_Os07g48010*, *LOC_Os07g48020*, and *LOC_Os02g14430*). Thus, the expressions of these DEGs were detected to validate the microarray results. According to the results, most of the DEGs were significantly up-regulated in OsHMGB707-OE lines than in WT plants (Figure 7B). Moreover, the expression of several DEGs was lower in *OsHMGB707* RNAi lines than in the WT plants. These results suggest that OsHMGB707 potentially regulates the expression of stress-related genes.

**FIGURE 8:**
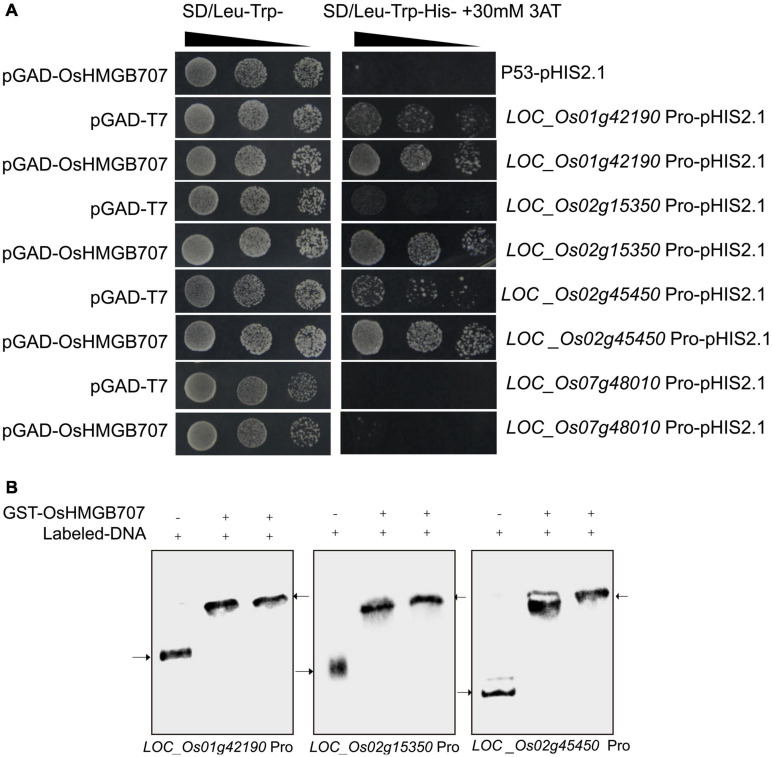
OsHMGB707 binds to the promoter region of target genes. **(A)** Yeast one-hybrid test. The upstream promoter regions were ligated into pHIS2.1 and transformed into yeast Y187 with pGAD-OsHMGB707. Pro, Promoter. pGAD-OsHMGB707 plus p53pHIS2.1 is negative control. **(B)** EMSA assay indicates that OsHMGB707 protein binds to the promoter DNA *in vitro*. The upper arrow shows the protein-DNA binding complex, while the lower arrow indicates the free double stranded DNA.

Further, the upstream promoter segments of several DEGs were cloned to investigate whether OsHMGB707 can directly regulate the proposed target genes. Yeast one-hybrid assays were used to test whether OsHMGB707 could bind these promoters. We found that yeast co-transformed with OsHMGB707 and pHIS2.1-promoters (i.e., *LOC_Os01g42190*, *LOC_Os02g15350*, and *LOC_Os02g45450*) constructs grew better than yeast containing pGAD-T7 plus pHIS2.1-promoters on the SD selective medium without histidine (Figure 8A). EMSA assays further displayed that OsHMGB707 binds to these promoter segments *in vitro* (Figure 8B). These results demonstrated that OsHMGB707 might bind to the promoter of the potential target genes.

## Discussion

### OsHMGB707 Positively Regulates Rice Drought Tolerance

Plants respond to drought stress in various ways, including changes in gene expression (Hirayama and Shinozaki, 2010). The HMG protein is an abundant non-histone chromosomal protein that modulates chromatin/DNA configuration, regulates gene transcription, and participates in DNA damage repair and plant innate immunity (Agresti and Bianchi, 2003; Choi et al., 2016; Charbonnel et al., 2018). Several studies have shown that some HMG proteins modulate stress response in Arabidopsis (Kwak et al., 2007; Lildballe et al., 2008; Mallik et al., 2020). Here, *OsHMGB707*, a novel HMGB gene in rice, was isolated and characterized. Our results revealed that *OsHMGB707* is localized in the rice drought QTL interval region, and its expression is remarkably induced by drought stress (Figure 2). Furthermore, *OsHMGB707* overexpression in rice significantly enhanced drought tolerance in transgenic rice (Figures 3, 5). These results indicate that *OsHMGB707* encodes a stress-induced HMG protein and positively regulates drought tolerance in rice.

In Arabidopsis, altering the expression of different HMGB proteins led to distinct phenotypes for stress tolerance. *AtHMGB1* overexpression reduced salt tolerance, whereas its knockout did not affect the salt tolerance level (Lildballe et al., 2008). Although overexpression of *HMGB5* did not affect salt and osmotic stress tolerance, plants lacking *HMGB5* showed reduced germination rate on media containing salt (Kwak et al., 2007). In this study, overexpression of *OsHMGB707* significantly enhanced drought stress tolerance (Figures 3, 5). Notably, the effects of *OsHMGB707* differed from the function of *AtHMGB1* and *HMGB5*. On the other hand, *OsHMGB707* knockdown did not significantly affect the stress tolerance of transgenic rice plants (Figures 4, 5). This finding concurs with the results reported in Arabidopsis that knocking out the *HMGB1* gene does not affect plant stress tolerance. Of note, this study examined RNAi knockdown lines but not knockout lines. Therefore, the residual activity of *OsHMBG707* in RNAi plants might still function. Considering that the rice genome contains six *HMGB* genes (Grasser et al., 2007), it is also possible that the redundant HMGB members have a compensation effect. Therefore, the knockout of all the six HMGB genes using the CRISPR/Cas9 technique would be more effective in characterizing their functions in rice.

### OsHMGB707 Encodes a Nuclear DNA Binding Protein

High mobility group proteins are often associated with chromatin/DNA, and many HMG proteins have been shown to localize in the cell nucleus. However, a few others localize in the cell cytosol and are even secreted out of the cell (Grasser et al., 2006; Pedersen et al., 2010). The different subcellular localization suggests that HMG proteins have different cellular functions (Pedersen et al., 2010). In this study, the OsHMGB707 protein was localized in the cell nucleus (Figure 6A), just like most HMG proteins. *In vitro*, EMSA methods have also demonstrated that many HMG proteins can bind DNA (e.g., four-way junction DNA) (Ritt et al., 1998; Wu et al., 2003; Grasser et al., 2004). Here, we found that OsHMGB707 protein could bind the four-way junction DNA and double-stranded target DNA (Figures 6C, 8), indicating that OsHMGB707 can bind different types of DNA. The DNA binding of most HMGB proteins lacks sequence specificity, is always scanned and is temporary (Launholt et al., 2006). However, some HMGBs can recognize specific DNA sequences and selectively bind to targets. For example, in maize, HMGB1 can recognize CCAAT *cis*-elements (Grasser et al., 1994). AtHMGB11 preferably binds to AT-rich DNA and shows structural bias for supercoiled DNA (Roy et al., 2016). DNA binding and footprinting assays further identified A(A/C)–ATA—(A/T)(A/T) as AtHMGB15 binding motif (Mallik et al., 2020). Here, OsHMGB707 could bind several target DNA (Figure 8), and several of these target promoters contain CCAAT elements (data not shown). However, it remains unknown whether OsHMGB707 recognizes specific DNA sequences.

### OsHMGB707 Regulates the Expression of Stress-Related Genes

Previous studies have shown that HMG proteins can bend DNA to facilitate the function of transcription regulatory factors and specific gene expressions (Bustin and Reeves, 1996; Bianchi and Agresti, 2005). In this study, the expression of several genes was up-regulated in *OsHMGB707*-overexpressing rice plants, including *LOC_Os02g45450* (*OsDREB1G*), *LOC_Os06g03670* (*OsDREB1C*), *LOC_Os02g15350* (*OsDOF7*), *LOC_Os01g42190* (encodes heat shock protein DnaJ), *LOC_Os01g04370* (encodes heat shock protein), and LOC_Os08g14190 (encodes flavonol sulfotransferase) (Supplementary Table 1). Some of these DEGs were also up-regulated by drought stress treatment (Figure 7A). Notably, some of these DEGs have been reported to be related to drought and other abiotic stresses. For example, DREB transcription factors play an important role in stress responses (Sakuma et al., 2006a,b). A previous study suggested that *OsDREB1G* and *OsDREB1C* may be involved in cold response in rice (Dubouzet et al., 2003). Heat shock proteins are responsible for protein homeostasis and tolerance to heat, drought, and other abiotic stresses (Jacob et al., 2017; Pulido and Leister, 2018). In plants, flavonol sulfotransferase-mediated sulfate-conjugation reactions play an essential role in plant adaptation to stress (Klein and Papenbrock, 2004).

The yeast one-hybrid test showed that OsHMGB707 directly binds to the promoter of *OsDREB1G* and several other target genes (Figure 8). The binding of OsHMGB707 to the promoter of the stress-related genes potentially enhances their expression enabling the plants to cope with drought stress. In animals and higher plants, HMG protein interacts with specific transcription factors to regulate gene expression (Grasser et al., 2007). For example, the maize HMGB1 protein can interact with DOF transcription factors to regulate tilling in maize seeds (Cavalar et al., 2003; Yamamoto et al., 2006). In this study, several transcription factors (e.g., OsDOF7 and OsDREB1C) were significantly induced in *OsHMGB707* OE plants. Cognizant of this, we speculated that OsHMGB707 might interact with these transcription factors and promote their expression. However, the yeast two-hybrid test indicated that OsHMGB707 does not interact with OsDOF7 and OsDREB1C transcription factors (data not shown). Therefore, the interaction between OsHMGB707 and transcription factors was not investigated further in this study. However, a detailed mechanism for OsHMGB707 in regulating the expression of target genes needs further investigation.

This study identified a novel drought-tolerance HMG gene in rice, *OsHMGB707*. Overall, the results of this study demonstrate that OsHMGB707 localizes in the nucleus where it may enhance the expression of stress-related genes, thereby positively modulating rice drought tolerance.

## Data Availability Statement

The original contributions presented in the study are publicly available. This data can be found here: the microarray data supporting the results of this article are available in NCBI Gene Expression Omnibus repository (http://www.ncbi.nlm.nih.gov/geo/) under accession numbers GSE130821 and GSE64576.

## Author Contributions

KX designed the experiments, performed the yeast assays, subcellular localization, stress treatments, physiological analysis, and wrote the manuscript. SC carried out the gene cloning and vector construction. TL performed the transformation of rice. HZ carried out the gene expression analysis. SY analyzed the microarray data. HL and LL supervised this work and assisted with editing the manuscript. All the authors read and approved the final manuscript.

## Conflict of Interest

The authors declare that the research was conducted in the absence of any commercial or financial relationships that could be construed as a potential conflict of interest.

## Publisher’s Note

All claims expressed in this article are solely those of the authors and do not necessarily represent those of their affiliated organizations, or those of the publisher, the editors and the reviewers. Any product that may be evaluated in this article, or claim that may be made by its manufacturer, is not guaranteed or endorsed by the publisher.

## Footnotes

1
https://biit.cs.ut.ee/clustvis/
