# Supplementary material for: Overexpression of OsHMGB707, a High Mobility Group Protein, Enhances Rice Drought Tolerance by Promoting Stress-Related Gene Expression
Source: Front Plant Sci. 2021 Aug 5;12:711271. doi: 10.3389/fpls.2021.711271 (PMC8375505; doi:10.3389/fpls.2021.711271)
Supplement: Supplementary file 1 [file Data_Sheet_1.DOCX]

**Supplement fig. S1**


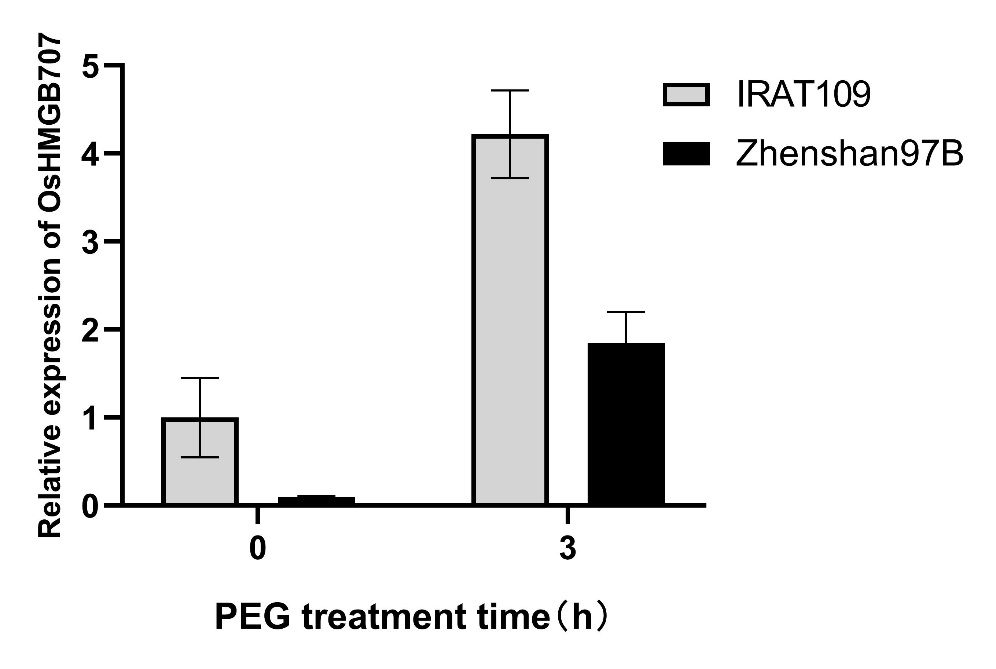


**Supplement Figure S1.** The expression of OsHMGB707 in IRAT109 and Zhenshan97B. The seedings of the two varieties were treated with 15%(m/V) PEG6000, and the leaves were sampled at 0 and 3 hours under PEG treatment. The expression of OsHMGB707 was detected through real-time qPCR. OsACT2 were used to be reference genes. Data represent mean±SE (n=3 biological replicates).

**Supplement fig. S2**


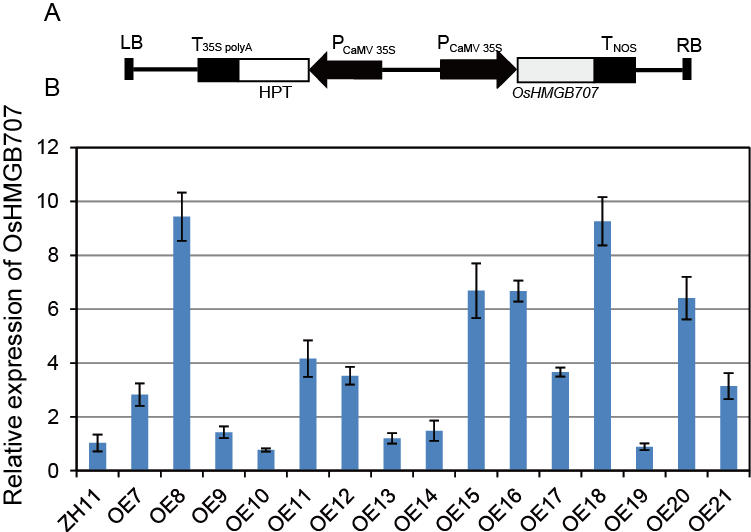


**Supplement Figure S2.** Overexpression of *OsHMGB707* in rice.

(A)The schematic structure of *OsHMGB707* overexpression construct. LB, left border; HPT, Hygromycin phosphotransferase; CaMV 35S, cauliflower mosaic virus 35S promoter; P, promoter; T, terminator; RB, right border. (B)The expression of *OsHMGB707* in overexpression transgenic rice lines detected through real-time qPCR. ZH11, wild type Zhonghua11 plants. OE7-21, *OsHMGB707* overexpression lines. *OsACT2* were used to be reference genes. Data represent mean±SE(n=3).

**Supplement Fig. S3**

**
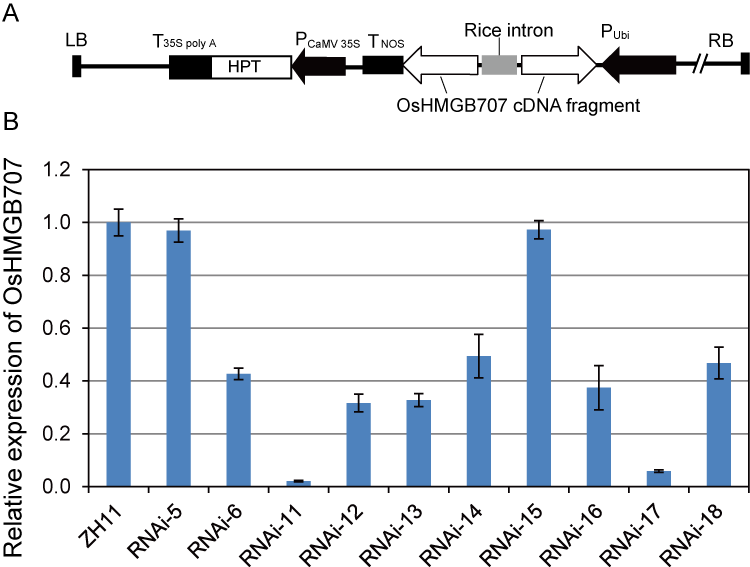
**

**Supplement Figure S3.** Suppression of *OsHMGB707* expression by RNAi.

(A)The schematic structure of *OsHMGB707* RNAi construct. LB, left border; HPT, Hygromycin phosphotransferase; CaMV 35S, cauliflower mosaic virus 35S; P, promoter; T, terminator; Ubi, ubiquitin; RB, right border. (B)The expression of *OsHMGB707* in RNAi transgenic rice lines detected through real-time qPCR. ZH11, wild type Zhonghua11 plants. RNAi5-6,11-18, *OsHMGB707* RNAi lines. *OsACT2* were used to be reference genes. Data represent mean±SE(n=3).

**Supplement Fig. S4**


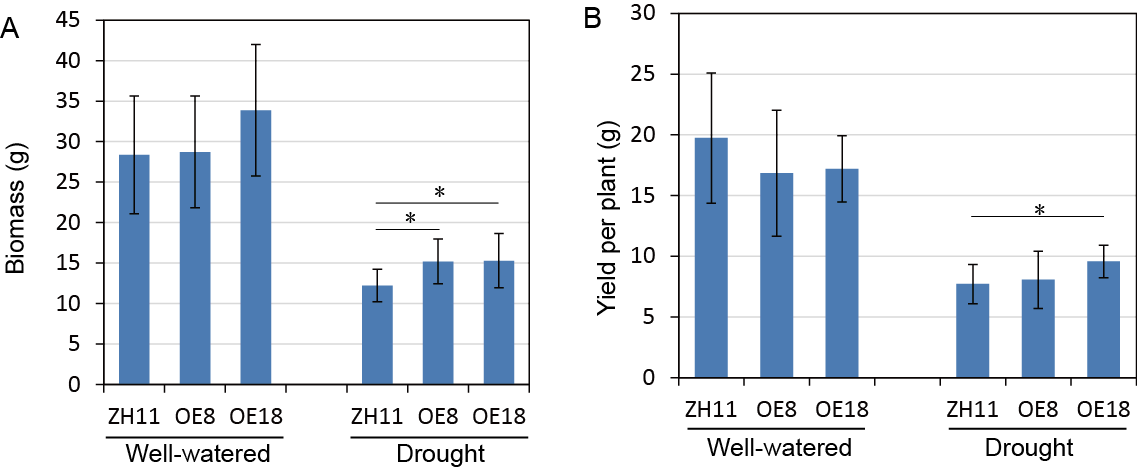


**Supplement Figure S4.** Repeated drought tolerance assessment of OsHMGB707 OE lines and WT rice plants under field conditions in Hainan 2018. (A) Biomass, (B) yield per plant in transgenic lines and WT rice plants. OsHMGB707-overexpression (OE) lines (OE8 and OE18) and WT rice plants (ZH11) were cultivated in the greenhouse, and irrigation was stopped before the heading stage. The water was re-supplied after approximately 30-day drought treatment. Data represents means ±SD (n=5-8), *p < 0.05, student’s t-test.

**Supplement Fig. S5**

**
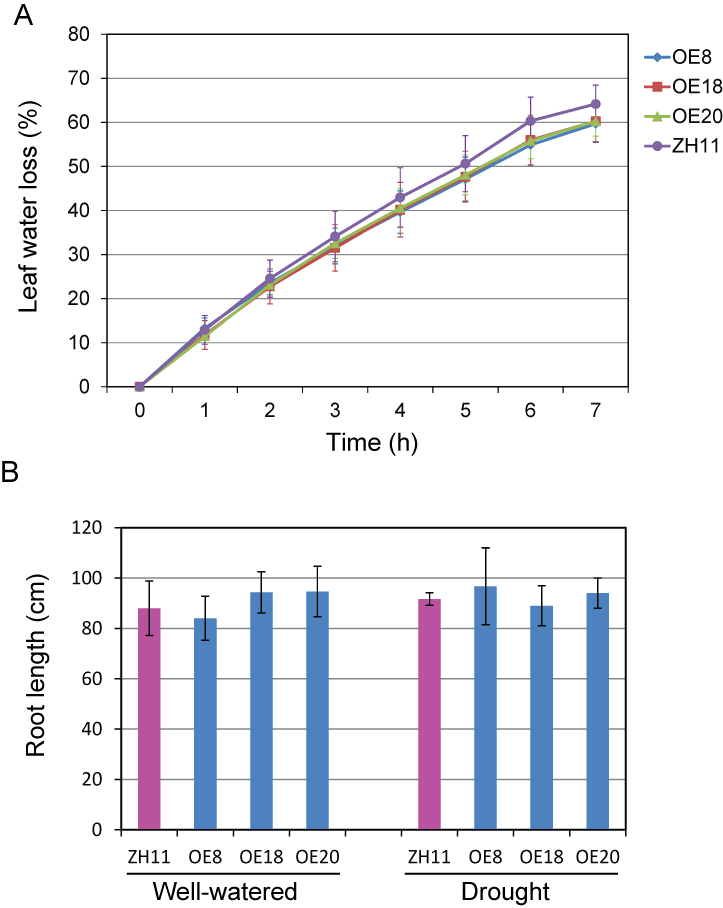
**

**Supplement Figure S5.** The leaf water loss rate (A) and the root length (B) of *OsHMGB707*-overexpressing rice plants. Data represent mean±SE (n=4 or 6).

**Supplement Fig. S6**


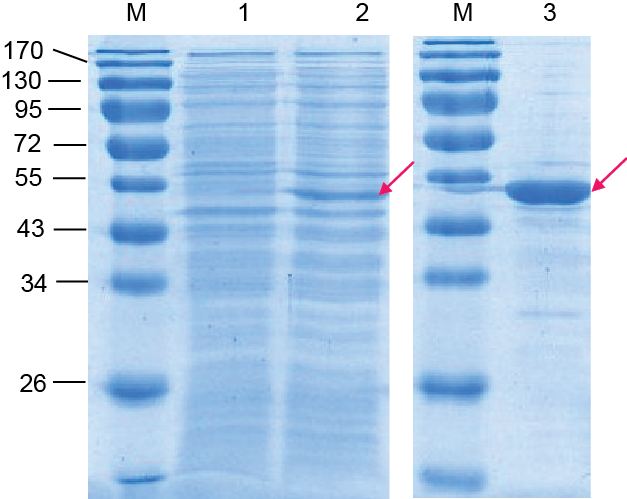


**Supplement Figure S6.** The induced expression and purification of GST-OsHMGB707 fusion proteins indicated by SDS-PAGE. M, protein molecular weight marker. 1, total protein before IPTG induction; 2, total protein after IPTG induction; 3, purified GST-OsHMGB707.
